# Apical Sealing Ability of MTA in Different Liquid to Powder Ratios and Packing Methods

**Published:** 2012-03-01

**Authors:** Ehsan Oraie, Amir Reza Ghassemi, Gholamhosein Eliasifar, Masoumeh Sadeghi, Arash Shahravan

**Affiliations:** 1. General Dentist, Kerman Oral and Dental Diseases Research Center, Kerman University of Medical Sciences, Kerman, Iran; 2. Department of Epidemiology, Kerman Oral and Dental diseases Research center, Dental School, Kerman University of Medical Sciences, Kerman, Iran; 3. Department of Endodontics, Kerman Oral and Dental diseases Research center, Dental School, Kerman University of Medical Sciences, Kerman, Iran

**Keywords:** Compound proportion, Concentration, Condensation, Dental, Leakage, Mineral trioxide aggregate, Retrofilling

## Abstract

**Introduction:**

In apical surgery, after apicoectomy and retro-preparation of canal, a retrofil material is applied to seal the apical region of the root canal. Mineral trioxide aggregate (MTA) is the gold standard material for this purpose. Changing water/powder ratios of MTA affects its properties. The purpose of this in vitro study was to determine the effect of liquid/powder ratio of retrofil MTA on apical dye leakage, and also compare two packing techniques for MTA.

**Materials And Methods:**

In this in vitro study, 126 intact single-root extracted teeth were instrumented using step-back technique, and obturated with lateral condensation method. The apical 3 mm of roots was resected, and retrograde preparation was performed by an ultrasonic device. Specimens were randomly assigned to 6 groups of 20 each and 6 teeth served as positive and negative controls. All teeth were retrofilled with White ProRoot MTA. Three groups were retrofilled with 0.28, 0.33, and 0.40 water/powder ratios of MTA and packed with plugger; the other three groups were retrofilled with 0.28, 0.33, and 0.40 water/powder ratios and packed with wet-cotton. Linear dye leakage was used to check apical sealing ability, and data were statistically analyzed using Kruskal-Wallis test.

**Results:**

In 0.40 liquid/powder ratio the best applicator for packing MTA was plugger. In 0.28 liquid/powder ratio, moist cotton pellet was the best applicator; in 0.33 liquid/powder ratio, there was no significant difference between the two techniques.

**Conclusion:**

Under the condition of this in vitro study, packing with moist cotton pellet in lower liquid/powder ratios of MTA, as well as packing with a plugger in higher liquid/powder ration decreased apical dye leakage.

## Introduction

In endodontic treatments, failure to achieve a well sealed root canal system is one of the most common causes of post-treatment diseases [1]. Apical surgery is usually indicated when routine RCT via coronal access cavity fails or is not feasible, and persistent contamination of the apical region occurs [2]. After apicoectomy and retro-preparation is conducted, a retrofil material is applied to seal the apical region of the root canal, to prohibit bacterial leakage [3].

Several materials and techniques have been advocated for retrofilling root canals. Among these materials, regarding plenty of in vitro and in vivo studies, mineral trioxide aggregate (MTA) stands out as the gold standard retrofil material for apical seal [2][4], due to its indispensable characteristics such as biocompatibility, non-toxicity, osteoinduction, cementogenesis [4][5]. It also provides a very good seal, has excellent marginal adaptation, maintains a high pH for a long period of time, and appears to induce a favorable tissue response [6][7][8].

MTA has some antibacterial and antifungal properties, depending on its powder/liquid ratio [5]. For example, different concentrations of white MTA have different effects on Candida albicans in vitro [9]. Increasing water/powder ratio increases solubility and porosity [10]. Totally, various water/powder ratios of MTA affects its properties [5]. Shahravan et al. reported water/powder ratios of MTA had no significant influence on the histological outcome of DPC on healthy human pulps [7]. To date, there is a little data about the impact of different concentrations of MTA on sealing ability.

One of MTA disadvantages is its poor handling characteristics [11][12]. Strength and hardness of MTA are affected by the condensation pressure during MTA placement. In an in vitro study, higher condensation pressure resulted in fewer voids and micro-channels and lower surface hardness values [13]. Expert clinicians have advocated two techniques for packing MTA in apical prepared cavity, i.e., applying pressure with moist cotton pellet and using pluggers. But there is no evidence of comparing these methods.

The purpose of this in vitro study was to determine the effect of liquid/powder ratio of retrofil MTA on apical dye leakage, and also a comparison on two packing techniques of MTA.

## Materials and Methods

In this in vitro study, 126 intact single-root canine and mandibular premolar extracted human teeth were collected. All teeth were free of restoration, former root canal therapy, and apical root resorption. All specimens were immersed in 5.25% NaOCl for two hours, in order to remove remnant soft tissues and debris. After adequate irrigation, root surfaces were assessed under a stereomicroscope (DM143, Motic Digital Microscope) with ×4.5 magnification. Detected cracks, carries, calcification, and any defect would exclude the tooth from the study. Three radiographs were taken at different stages of specimen preparation: on tooth selection, during endodontic treatment, and after obturation.

Coronal access cavities were prepared by a high-speed handpiece and diamond burs, under continuous air/water spray. Working lengths were visually determined by subtracting 0.5 mm from the length of a size 15 K-file (Dentsply Maillefer, Ballaigues, Switzerland) at the apical foramen. Instrumentation with K-files was performed to the working length up to file #40 as the master apical file (MAF), and continued up to file #80 with step-back technique. Canals were irrigated by 5.25% hypochlorite sodium during canal preparation. After drying canals with paper points (Ariadent, Tehran, Iran), all were obturated with gutta-percha (Ariadent, Tehran, Iran) and AH26 sealer (Dentsply, Konstanz, Germany), using lateral condensation technique.

Crowns were sectioned at the cementoenamel junction. After removing 3 mm of coronal gutta-percha, the access cavities were filled by temporary filling (Cavizol, Golchai, Tehran, Iran). Root end resection was performed by cutting 3 mm of the apex prependiculafr to the long axis of the root using high-speed handpiece and fissure burs. An ultrasonic device (NSK-Nakanishi Inc., Tokyo, Japan) with ultrasonic retro tip E32D (NSK-Nakanishi Inc., Tokyo, Japan) was used to prepare the apical cavities 3 mm deep. Feather-like back and forth motion was used for cutting with the ultrasonic tips, which were enveloped in water spray [14].

Specimens were randomly assigned to 6 groups (n=20). All were retrofilled with White ProRoot MTA (Dentsply, Tulsa Dental, USA). In groups A, B, and C MTA was packed into apical cavity using an endodontic plugger (Dentsply Maillefer, Ballaiques, Switzerland), and liquid/powder ratios were 0.28, 0.33, and 0.40, respectively. In groups D, E, and F MTA was packed into apical cavity using a moist cotton pellet, and liquid/powder ratios were 0.28, 0.33 and 0.40, respectively. Three teeth served as positive control, in which, warm gutta-percha without any sealer was packed in apical cavities. Three teeth served as negative control, which were retrofilled with 0.33 liquid/powder ratio of MTA. Apical end was sealed by sticky wax.

For obtaining appropriate liquid/powder ratios of MTA, a digital scale (GF-300, A&D Company, Tokyo, Japan) with accuracy of 0.001 gram was used.

Specimens were incubated at 37ºC and 100% relative humidity. After seven days of incubation, paper points were used to ensure MTA had been set properly in all roots. If improper adaptation and integrity defects were detected in a root, MTA was removed from apical cavity and new MTA was inserted, regarding each group instructions, and followed by seven days of incubation. The roots were surface coated with two layers of nail varnish. The varnish was applied onto the entire root surface, except for the area corresponding to the resected apical surface.

**Figure 1:**
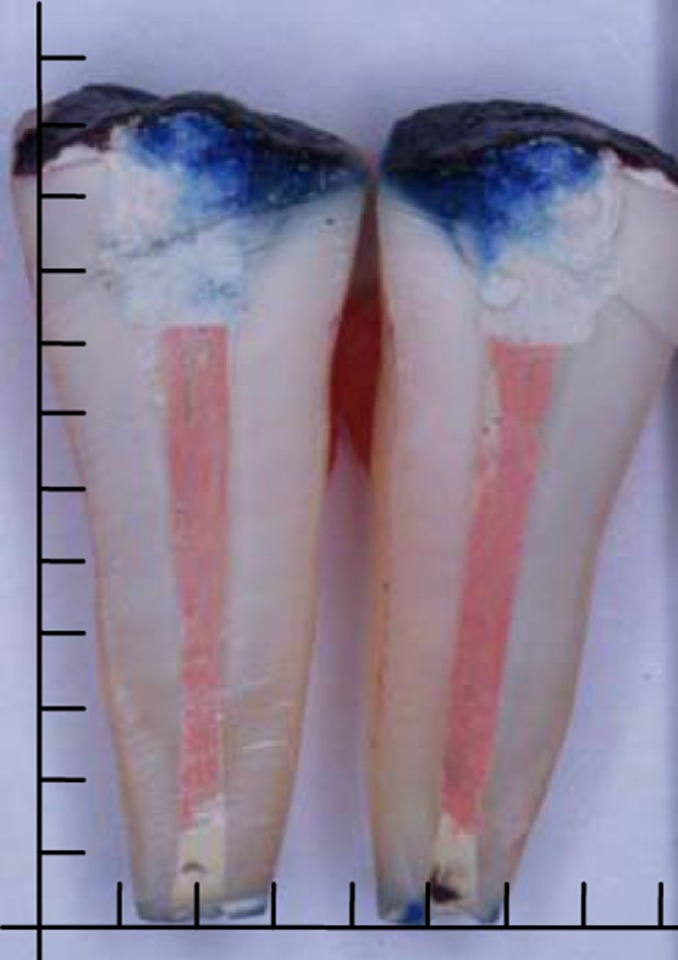
Photograph of a sectioned tooth after dye penetration (the distance between two lines represents 2 mm)

For dye penetration, the specimens were immersed in 2% methylene blue for seven days [15]. Then, after adequate flushing, the varnish and sticky wax coatings were removed with a scalpel blade, in order to prohibit any color artifact during cutting process with the diamond disk (D&Z, Berlin, Germany). Longitudinal cuts in mesio-distal direction, approximately in mid-root, and passing through the apex, split each root into two half-roots. All the procedures were performed under continuous irrigation with water. A digital camera (Olympus, DX8R, Japan) was used to take high resolution photographs [4][16]. Two experienced independent examiners calibrated for the technique and blinded to the groups, evaluated the photographs on a computer monitor. The highest leakage values reported by the examiners were selected in each specimen. The mean penetration depth of two half-roots was recorded for each tooth (Figure 1).

For each group a mean value of penetration depth and standard deviation were calculated. The results were analyzed by Kruskal-Wallis Test. To compare mean values between the groups the Mann-Whitney U Test was performed. All statistical analyses were carried out at 5% significance level, using the Statistical Package for the Social Sciences (SPSS) version 17.0 for Windows.

## Results

The negative control samples showed no dye penetration, while the positive controls showed complete leakage. Seven specimens of group D, 6 specimens of group E, and 2 specimens of group F were excluded from the study, because of insufficient removal of nail varnish before root sectioning and subsequent dye artifacts.

Kruskal-Wallis test indicated a statistically significant variation between the groups in which MTA was packed with plugger (P<0.001). In this technique, group A had the maximum average of dye penetration compared to the other groups (Table 1). Statistically significant difference was observed between groups B and C, and also between groups A and C. Among all specimens of plugger technique, those with 0.40 liquid/powder ratio showed the lowest dye penetration. Among those groups in which MTA was packed with moist cotton pellet, a statistically significant variation was observed as well (P<0.001). In this technique, significant difference in dye penetration was found only between groups E and F. Among all groups of moist cotton pellet technique, the 0.28 liquid/powder ratio had the lowest dye leakage (Table 1).

**Table 1:** Mean±SD of dye penetration in all experimental groups

**Packing Technique**	**Experimental Groups**	**N**	**Mean(SD) **
	A- 0.28	20	0.845(0.69)
Plugger	B- 0.33	20	0.431(0.54)
	C- 0.40	20	0.0(0.0)
**Moist cotton pellet**	**Experimental Groups**	**N**	**Mean(SD)**
	D- 0.28	13	0.134(0.33)
	E- 0.33	14	0.62(0.97)
	F- 0.40	18	0.37(0.75)

Mann-Whitney U Test was used to compare dye penetration means between those groups with similar liquid/powder ratios and different packing methods. In 0.28 liquid to powder ratio, a significant lower leakage was observed in plugger technique (P=0.01). In 0.40 liquid/powder ratio moist cotton technique significantly showed lower leakage (P=0.05); and in 0.33 liquid/powder ratio there was no significant difference between the two techniques (P>0.4).

## Discussion

Achieving a well sealed root canal system is one of the most important aims of endodontics [1]. Apical surgery is indicated in the cases of reinfection or persistent contamination after routine RCT [2]. This procedure consists of apicoectomy and retrograde preparation/filling [3]. Obtaining a secure seal is one of the main purposes in retrofilling. Seal of MTA is influenced by various factors such as thickness of the dentinal wall, the dye pH, the type of dye, pretreatment with chelating agents, the tooth storage environment before the experiment, and the setting status of MTA before its placement in the dye [4]. Apical seal can be assessed by dye penetration test [5]. If the leakage of small molecules of dye (tracer solutions) is prevented by retrofilling materials, the infiltration of larger substances (such as bacteria and their products) can be prevented as well [14]. Simplicity and cheapness are other reasons to select this test, but this method has some shortcomings such as providing semi-quantitative results and yielding a high level of variation [17][18].

In the present study, we tested 0.28, 0.33 and 0.40 water/powder ratios of MTA, which were similarly used in Fridland and Rosado [10] and Shahravan [7] studies, in order to allow direct comparisons with their findings. The 0.33 ratio is usually suggested by the manufacturer. The current study revealed that liquid/powder radio of retrofil MTA was an important factor in apical seal of the specimens. In the groups of plugger technique, those with 0.28 liquid/powder ratio showed the lowest dye penetration; while, among the groups of moist cotton pellet technique, the 0.40 liquid/powder ratio had the lowest dye leakage. On the other hand, in 0.28 liquid/powder ratio, in order to obtain the lowest leakage, the best applicator for packing MTA was plugger. In 0.40 liquid/powder ratio moist cotton pellet was the best applicator; and in 0.33 liquid/powder ratio there was no significant difference between the two techniques (P>0.4). These two techniques are suggested by the manufacturer for packing MTA in retrograde filling, but there was no comparison on these two techniques so far. These results can be construed rational, considering this fact that MTA requires wetness for its setting process. Dicalcium silicate, tricalcium silicate, and bismuth oxide are the main components of MTA. Wet environment gradually strengthens MTA because dicalcium silicate hydration rate is slower than tricalcium silicate rate; therefore, in lower liquid/powder ratios moist cotton pellet is more effective, because additional wetness from cotton helps setting process [5].

According to Al-Hezaimi et al. study, lowering the powder/liquid ratio might adversely affect antifungal properties of MTA [9]. Fridland et al. reported that solubility and porosity of MTA increases in higher water/powder ratios [10]. In contrast, Shahravan et al. evaluated histological outcome of direct pulp capping with MTA in different water/powder ratios. They found no significant difference in the diameter, morphology and continuity of the calcified bridge, intensity and type of inflammation or presence of necrosis in human dental pulp [7].

## Conclusion

Under the condition of this in vitro study, using a plugger or moist cotton pellet for packing MTA retrofil material did not make significant difference in apical dye leakage. In order to obtain the lowest dye leakage, the best applicator for packing MTA is a plugger with 0.28 liquid/powder ratio, and moist cotton pellet when mixing MTA with 0.40 liquid/powder ratio. Further randomized clinical trials or at least animal models are needed to confirm the findings of this in vitro study. In addition, more accurate leakage tests such as fluid filtration can provide us with more reliable results.
